# Cardiovascular autonomic function in middle-aged people with long-term cervical and upper thoracic spinal cord injuries

**DOI:** 10.1080/10790268.2024.2403791

**Published:** 2024-10-11

**Authors:** Mattias Hill, Sophie Jörgensen, Gunnar Engström, Margaretha Persson, Pyotr G. Platonov, Viktor Hamrefors, Jan Lexell

**Affiliations:** 1 Department of Health Sciences, Lund University, Lund, Sweden; 2 Department of Rehabilitation Medicine, Skåne University Hospital, Lund, Sweden; 3 Department of Clinical Sciences in Malmö, Clinical Research Centre, Lund University, Malmö, Sweden; 4 Department of Internal Medicine, Skåne University Hospital, Malmö, Sweden; 5 Department of Cardiology, Clinical Sciences, Lund University, Lund, Sweden; 6 Department of Cardiology, Skåne University Hospital, Malmö, Sweden

**Keywords:** Autonomic nervous system, Cardiovascular diseases, Spinal cord diseases

## Abstract

**Objectives:**

To examine cardiovascular autonomic function in middle-aged people with long-term cervical and upper thoracic spinal cord injury (SCI) compared with the general population, and explore if the neurological level of injury (NLI) is related to cardiovascular autonomic function.

**Design:**

Population-based cross-sectional study with matched controls.

**Setting:**

Outpatient SCI unit in Southern Sweden.

**Participants:**

Twenty-five individuals (20% women, mean age 58 years and mean time since injury 28 years, NLI C2-T6, American Spinal Injury Association Impairment Scale A-C) from the Swedish SPinal Cord Injury Study on Cardiopulmonary and Autonomic Impairment (SPICA). Matched controls were obtained from the population-based Swedish CArdioPulmonary bioImage Study (SCAPIS) at a ratio of 5:1.

**Interventions:**

Not applicable.

**Outcome measures:**

24 h electrocardiography and deep breathing tests. 24 h ambulatory blood pressure (BP) monitoring and orthostatic BP tests.

**Results:**

In individuals with SCI compared with controls, heart rate variability (24h mean SD of the normal-to-normal interval 112 ms vs 145 ms, P < 0.001) and diastolic orthostatic BP increase (2.0 and 9.4 mmHg, P < 0.001), were significantly lower, whereas BP variability was significantly higher (24h mean systolic SD_BP_ 17.8 mmHg vs 15.7 mmHg, P = 0.029). Circadian patterns of heart rate variability and BP (lack of nocturnal dip) were significantly different among the individuals with SCI than controls. Higher NLI was significantly (P < 0.05) correlated with impairments to various cardiovascular autonomic function variables.

**Conclusions:**

This exploratory study indicates that cardiovascular autonomic function is impaired in middle-aged people with long-term cervical and upper thoracic SCI compared with the general non-SCI population, and more pronounced with a higher NLI. Future research is needed to understand the pathophysiological mechanisms underlying these impairments, and the prognostic significance for individuals with SCI.

**Trial registration:**

ClinicalTrials.gov identifier: NCT03515122.

## Introduction

Cardiovascular disease (CVD) is one of the leading causes of death in people with spinal cord injury (SCI) (1). Cardiovascular autonomic functional impairments are important risk markers for CVD (2). People with cervical and upper thoracic SCI may have lifelong cardiovascular disturbances, due to loss of supraspinal control of the sympathetic outflow to the heart and the vasculature (3). The cardiovascular impairments could manifest as low resting blood pressure (BP) and low heart rate (HR), and daily fluctuations in BP due to orthostatic hypotension and autonomic dysreflexia (AD) (3). Indeed, daily symptoms of hypotension and AD in this population have been reported to be 76% and 68%, respectively (4). These manifestations have social, emotional, and physical implications on daily life of people with SCI, in terms of experiencing symptoms and a lack of awareness of how to administer health care in general medical situations (5).

The autonomic modulation of the cardiovascular system can be examined with electrocardiography to assess the heart rate variability (HRV) and thereby reveal sympathetic and parasympathetic activity (2,6). Time domain and frequency domain analyses of HRV are frequently used (6). Time domain values are the amount of variability in measurements of the time period between successive heart beats (*i.e.* changes over time), whereas frequency domain values are calculations of the distribution of the amount of signal energy into different frequency bands (6). The parasympathetic (cardiovagal) system is mainly responsible for the quick adjustments of HR. This could be assessed using a deep breathing test (DBT) to compare the HR during inspiration and expiration. DBT is a short-term recording during paced breathing when the cardiovagal activity will be the primary source of the HRV (2,6). Cardiovagal activity can also be assessed by analysis of the root mean square of successive differences (RMSSD), and the high-frequency spectrum (HF) of HRV (2,6). The sympathetic part of the autonomic nervous system has a slower response action and is partly reflected in long-term (24h) recordings by the standard deviation of the NN interval (SDNN) and low frequency (LF) variations of HRV (6). The LF/HF ratio is sometimes used as a measure of the balance between the sympathetic and parasympathetic systems (6). Ambulatory and orthostatic BP can be used to measure BP variability (BPV), circadian patterns, and orthostatic responses (7) and thereby assess autonomic BP regulation.

Impairments of cardiovascular autonomic function are common in people with cervical and upper thoracic SCI (3). The relationship between a higher neurological level of injury (NLI) and a lower systolic and diastolic BP, and a lower BP in the seated compared with the supine position have been identified (8). However, studies comprehensively assessing both cardiac and vascular autonomic functions in long-term SCI, and comparing with matched controls are scarce (3,9–11).

To address this knowledge gap, we initiated the Swedish SPinal Cord Injury Study on Cardiopulmonary and Autonomic Impairment (SPICA) (4). SPICA is a broad and in-depth assessment of the cardiopulmonary systems including cardiovascular autonomic function in 25 middle-aged individuals with long-term cervical and upper thoracic SCI and sex and age-matched non-SCI controls. In previous studies, we have presented the study design and initial results, functional and structural impairments of the pulmonary system, and atherosclerosis (4,12,13).

The objectives of the present study are to: (1) describe cardiovascular autonomic function in middle-aged individuals with long-term cervical and upper thoracic SCI; (2) determine whether cardiovascular functions are impaired compared with the general population; and (3) explore if the NLI is related to cardiovascular autonomic function. We hypothesize that cardiovascular autonomic function is significantly impaired among the individuals with SCI compared with the controls, and that a higher NLI is related to impairments of cardiovascular autonomic function. We hypothesize that these differences will be identified in measures assessing sympathetic modulation of the HR and BP, *i.e.* SDNN, LF, orthostatic responses and BPV.

## Materials and methods

### Design

The present study is part of the Swedish SPinal Cord Injury Study on Cardiopulmonary and Autonomic Impairment (SPICA) (4). SPICA is a population-based cross-sectional study with matched controls from the general population. SPICA conforms to the STROBE (Strengthening the Reporting of Observational Studies in Epidemiology) guidelines (14) and is registered at ClinicalTrials.gov (Identifier: NCT03515122; Registration date: 2 May 2018).

SPICA is based on the Swedish CArdioPulmonary and bioImage Study (SCAPIS) (15). SCAPIS comprises a cohort of 30,154 individuals from the general population aged 50–64 years. The aims of SCAPIS are to study disease mechanisms and improve risk prediction of chronic obstructive pulmonary disease and CVD. Control data from SCAPIS are used to compare with the data of SPICA.

### Statement of ethics

SPICA was approved by the Regional Ethical Review Board in Lund, Sweden (No. 2017/756), and we have followed the Declaration of Helsinki for research on humans (4). All participants signed a written informed consent form prior to enrollment. All participants received written and oral information of their study findings at the end of the study. Pathological findings were managed to establish further investigations or treatment.

### SPICA participants

Inclusion criteria in SPICA were: (i) traumatic SCI, American Spinal Injury Association Impairment Scale (AIS) A-C (16), NLI C1-T6; (ii) time since injury >5 years; (iii) age 50–65 years (iv) resident in the Skåne region, Sweden. Ventilator dependency was an exclusion criterion due to the inability to take part in several of the examinations. There were no cardiovascular exclusion criteria as the study objective of SPICA was to explore cardiopulmonary health in a population-based sample of individuals with cervical and upper thoracic SCI.

The participants were recruited from the SCI unit at the Department of Rehabilitation Medicine, Skåne University Hospital in Lund, Sweden. The SCI unit is responsible for the lifelong follow-up of all people with SCI in southern Sweden. The Skåne region (total population of about 1.3 million people) comprises part of the catchment area.

No power calculation was made in SPICA, as the study design is exploratory and descriptive. There were 38 eligible individuals and 25 consented to participate. There were no significant differences regarding sex or age, injury characteristics (NLI, AIS grade, age at injury and time since injury) between the 25 participants and the 13 non-participants (4).

### Controls

In total, 6,251 individuals from the general population were enrolled during 2014 to 2018 at the Malmö study site of SCAPIS (2). Matched control data at a ratio of 5:1 were obtained from SCAPIS individuals who had performed the 24h assessments (described below). Controls were assessed by the same staff and during the same period (one year) as the individuals in SPICA. The controls were matched for sex and age (±2 years). If multiple controls were available to match an individual with SCI, controls were chosen at random. Data access and matching, including randomly matching of controls, were performed independently by SCAPIS after an application to the SCAPIS Data Mart (https://scapisdata.wlab.gu.se/Project/Index). The data were applied for and then made available for the researchers.

Control data were not complete for all variables, in particular DBT data, as these were available in 4,654 of the 6,251 SCAPIS participants (2). Missing control data never exceeded two controls per individual with SCI except in two cases of the DBT.

### Data collection

In Table 1, an overview and description of the assessments and definitions in SPICA of relevance for the present study are reported. All data collection was performed on two separate occasions during the period January to May 2018 at the SCAPIS study site at Skåne University Hospital in Malmö, Sweden. The first author participated in all the data collection procedures.

**Table 1:** Cardiovascular functional assessments and definitions.

Assessments	Definitions of parameters
Electrocardiogram	
Heart rate variability (HRV) (6)	The HRV is the changes in the time intervals occurring between consecutive heartbeats. HRV can be measured by different methods. Commonly used methods are time domain and frequency domain methods. Time domain measures evaluate the amount of variance of the changes in the time intervals. Frequency domain measures evaluate the amount of power in different frequency bands (high, low, very low and ultralow frequencies). These oscillations of the heart rhythm tend to correlate to different physiological phenomenon.
Time domain	
Heart rate (HR) (bpm)	Heart beats per minute
Expiration–inhalation difference (E-I) (bpm)	The difference in HR between expiration and inspiration measuring cardiovagal function
Standard deviation of heart rate (SD_HR_) (bpm)	The standard deviation of the HR
Mean circular resultant (MCR)	A vector-based measure in the assessment of HRV reducing the effects of differences between subjects in mean heart rate, and premature ventricular contractions (2)
Root mean square of successive differences (RMSSD) (ms)	Used to estimate changes in HRV mediated from parasympathetic activity and reflects the beat-to-beat variance in heart rate. RMSSD measures are obtained by calculating time differences between successive heartbeats. Thereafter each value is squared and averaged. Finally, the square root of the total is obtained. RMSSD primarily measures cardiovagal function.
Standard deviation of the NN interval (SDNN) (ms)	Corresponds to all intervals between adjacent QRS complexes and measures both sympathetic and parasympathetic function
Frequency domain	
High frequency (HF) (ms^2^)	The power in HF range (0.15-0.4 Hz) corresponding to heart rate variations in relation to the respiratory cycle reflecting parasympathetic activity
Low frequency (LF) (ms^2^)	The power in LF range (0.04-0.15 Hz) is due to both cardiac parasympathetic and sympathetic activity, and to a smaller proportion, other factors such as the renin-angiotensin-aldosterone system
LF/HF	Suggested to measure the balance between cardiac sympathetic (LF) and parasympathetic (HF) activity
Blood pressure	
Systolic blood pressure (mmHg)	The pressure of the arterial walls during the heart beat
Diastolic blood pressure (mmHg)	The pressure of the arterial walls in between heart beats
Orthostatic blood pressure^a^ (mmHg)	The change in blood pressure between lying down resting and three minutes after rising up to a seated or standing position
Orthostatic hypotension	Orthostatic hypotension was defined as a drop in systolic BP ≥20 mmHg or diastolic BP ≥10 mmHg after 3 min of body position change from supine to seating (individuals with SCI) or standing (controls) (18)
Ambulatory blood pressure	Blood pressure measurements continuously over a 24-hour period
Standard deviation of blood pressure (SD_BP_) (mmHg)	The standard deviation of the ambulatory blood pressure measurements
Circadian patterns	
Dipper	10–20% nocturnal systolic blood pressure fall
Extreme-dipper	>20% nocturnal systolic blood pressure fall
Non-dipper	0-10% nocturnal systolic blood pressure fall
Reverse-dipper	Nocturnal systolic blood pressure rise

Abbreviations: NN, normal-to-normal.

^a^ Seated position for the individuals with SCI and standing for the controls.

#### Characteristics

Data on demographics, injury characteristics and medical history were obtained using a study-specific questionnaire, reviewing medical records, interviewing the individuals with SCI and examining them clinically.

Body weight was recorded using a portable scale for wheelchairs (Corina Medical MPWS 300; Rörvik, Sävsjö, Sweden). Body height and waist circumference (WC) were measured in a supine position with a flexible measuring tape. Body mass index (BMI) was calculated from the height and weight measures.

Corresponding matched control data were obtained from the SCAPIS study-specific questionnaire and anthropometry data collected in SCAPIS as previously reported (15).

#### Cardiovascular assessments

The cardiovascular assessments were all part of the SCAPIS baseline protocol at the Malmö study site and used to assess individuals with SCI according to the protocol. Consequently, the SPICA study adhered to the SCAPIS methodology.

***Electrocardiogram*.** DBT and 24h Holter were performed to assess HRV. DBT was performed as previously described in SCAPIS (2), with a 12-lead electrocardiogram (ECG) recording in the supine position after a 5-minute rest while the individuals were taking long breaths for one minute (Cardiax PC-ECG, Mesa, Benediktbeuern, Germany). The individuals were instructed to breathe through the nose, if possible, and inhale and exhale each time over a period of 5 s. ECGs were visually scanned before inclusion (2) and thereafter analyzed according to Löllgen *et al.* (17). Six HRV measures were calculated: the median-based expiration–inhalation difference (E-I_median_), mean-based expiration–inhalation difference (E-I_mean_), expiration–inhalation ratio (E/I), standard deviation of heart rate (SD_HR_), mean circular resultant (MCR) and RMSSD (cf. Table 1).

ECG signal analysis over a period of 24h (Holter) was recorded (EC-3H/ABP, Labtech, Debrecen, Hungary) with a sampling frequency of 256 Hz analyzed (Cardiospy®, Labtech, Debrecen, Hungary) and with a standard 3-lead placement. The skin was cleaned, and hair was removed before applying the electrodes. The 24h ECGs were scrutinized by a trained physician to remove artefacts and validate findings. Five measures of HRV were calculated using the Cardiospy software (v 5.4.1.0) (Task force of the European Society of Cardiology): SDNN, RMSSD, HF, LF and LF/HF ratio (cf. Table 1).

Atrial fibrillation occurred in one individual with SCI during the 24h registration and these data were excluded due to the substantial distortion of the HRV assessment.

***Blood pressure*.** Measures of resting systolic and diastolic BP were made twice in each arm with an automatic device after a 5-minute rest (Omron M10-IT, Omron Health care Co, Kyoto, Japan).

Orthostatic BP (Omron M10-IT) was measured twice in the supine position after a 5-minute rest, with one minute between the measurements. The individual then raised (with or without assistance) to a seated position and instructed to be still and not talk for three minutes. The matched controls were instructed to rise to a standing position. After three minutes another two BP measurements were performed with no pause between the measurements. The mean measures were used to calculate the difference in BP (*i.e.* subtracting the supine BP from standing or seated BP). Orthostatic hypotension was defined as a drop in systolic BP ≥20 mmHg or diastolic BP ≥10 mmHg 3 min after body position change from supine to seating (individuals with SCI) or standing (controls) (18).

Ambulatory 24h BP (ABPM) measurements were taken every 30 min for 24h to assess BPV and circadian BP patterns (EC-3H/ABP). Data from one individual with SCI were excluded due to several invalid measures.

### Statistical analysis

Data are presented using quantitative descriptive statistics with mean, standard deviation, median, minimum and maximum, and frequency and proportion (%), where appropriate.

The individuals with SCI and the controls were compared using conditional logistic regression. Highly skewed variables (pack years, HF_night_ and LF_day_, cf. Table 1) were log-transformed for analysis. If data were missing, corresponding control data were excluded. A supplementary one-sided paired samples t-test was computed in the individuals with SCI to compare the mean difference between day and night LF/HF ratios (cf. Table 1) due to the initial results from the conditional logistic regression.

Spearman rank correlations were computed to explore associations between NLI (defined as each higher NLI C1-T6) and cardiovascular autonomic function and only presented if statistically significant (P values less than 0.05).

The statistical analyses were performed using the IBM SPSS Statistics Software v 28 (IBM Corporation, Armonk, NY, USA).

## Results

### Characteristics of the individuals with SCI and controls

In Table 2, the characteristics of the 25 individuals with SCI and the 125 controls from the general population are presented.

**Table 2:** Characteristics of the 25 individuals with spinal cord injury and the 125 matched controls from the general population.

	Individuals with SCI	Matched controls	OR^a^ (95% CI)	P value
Sex (n (%))				
Women	5 (20)	25 (20)		
Men	20 (80)	100 (80)		
Age (years) (mean ±SD; median, min-max)	58 ±5; 58, 50–65	58 ±4; 57, 50–65	0.84 (0.49–1.5)	0.54
Age at injury (years) (mean ±SD; median, min-max)	30 ±12; 28, 7–56			
Time since injury (years) (mean ±SD; median, min-max)	28 ±13; 31, 6–53			
Injury level (n (%))				
C1-C4	10 (40)			
C5-C8	6 (24)			
T1-T6	9 (36)			
Severity of injury (n (%))				
AIS A	15 (60)			
AIS B	8 (32)			
AIS C	2 (8)			
Medical history (n (%); mean ±SD; median, min-max)				
Smoking status				
Current regular smoker	2 (8)	19 (16)	0.48 (0.11–2.2)	0.35
Pack years^b,c^	8 ±11; 4, 0–46	17 ±30; 2, 0–154		0.66
Previous cardiovascular event	2 (8)	4 (4)		
Myocardial infarction	2 (8)	3 (3)		
Stroke	0 (0)	1 (1)		
Atrial fibrillation	3 (12)	4 (4)		
Diabetes mellitus	4 (16)	5 (4)		
Antihypertensive medications	5 (20)	23 (18)		
Anthropometry (mean ±SD; median, min-max)				
BMI; kg/m^2^	25.1 ±5.3; 25.4, 14.1–34.4	27.3 ±4.2; 26.7, 15.4–37.6	0.88 (0.79–0.99)	**0.032**
Waist circumference (cm)	101 ±14; 103, 70–127	98 ±13; 99, 63–130	1.0 (0.99–1.06)	0.23

Conditional logistic regression analyses of differences between individuals with SCI and controls.

Abbreviations: AIS, American Spinal Injury Association (ASIA) Impairment Scale (16); SCI, spinal cord injury.

^a^ The ORs of the continuous variables implicate the odds of being an individual with SCI for a one-unit increase in the variable.

^b^ One pack year is defined as smoking 20 cigarettes (or equivalent consumption of cigar, pipe) every day for a whole year.

^c^ Conditional logistic regression computed on transformed data because of highly skewed data.

The frequency of current smokers among the individuals with SCI and controls was 8% and 16%; *P* = 0.35, respectively. Among the individuals with SCI and controls, the proportions with previous CVD events were 8% and 4%, diabetes mellitus 16% and 4%, and use of antihypertensive agents 20% and 18%, respectively.

BMI was significantly lower among the individuals with SCI than the controls (25.1, ±5.3 vs 27.3, ±4.2; *P* = 0.032), whereas WC was comparable (101, ±14 vs 98, ±13 cm; *P* = 0.23).

### Cardiovascular autonomic function in individuals with SCI and controls

**Figure 1:**
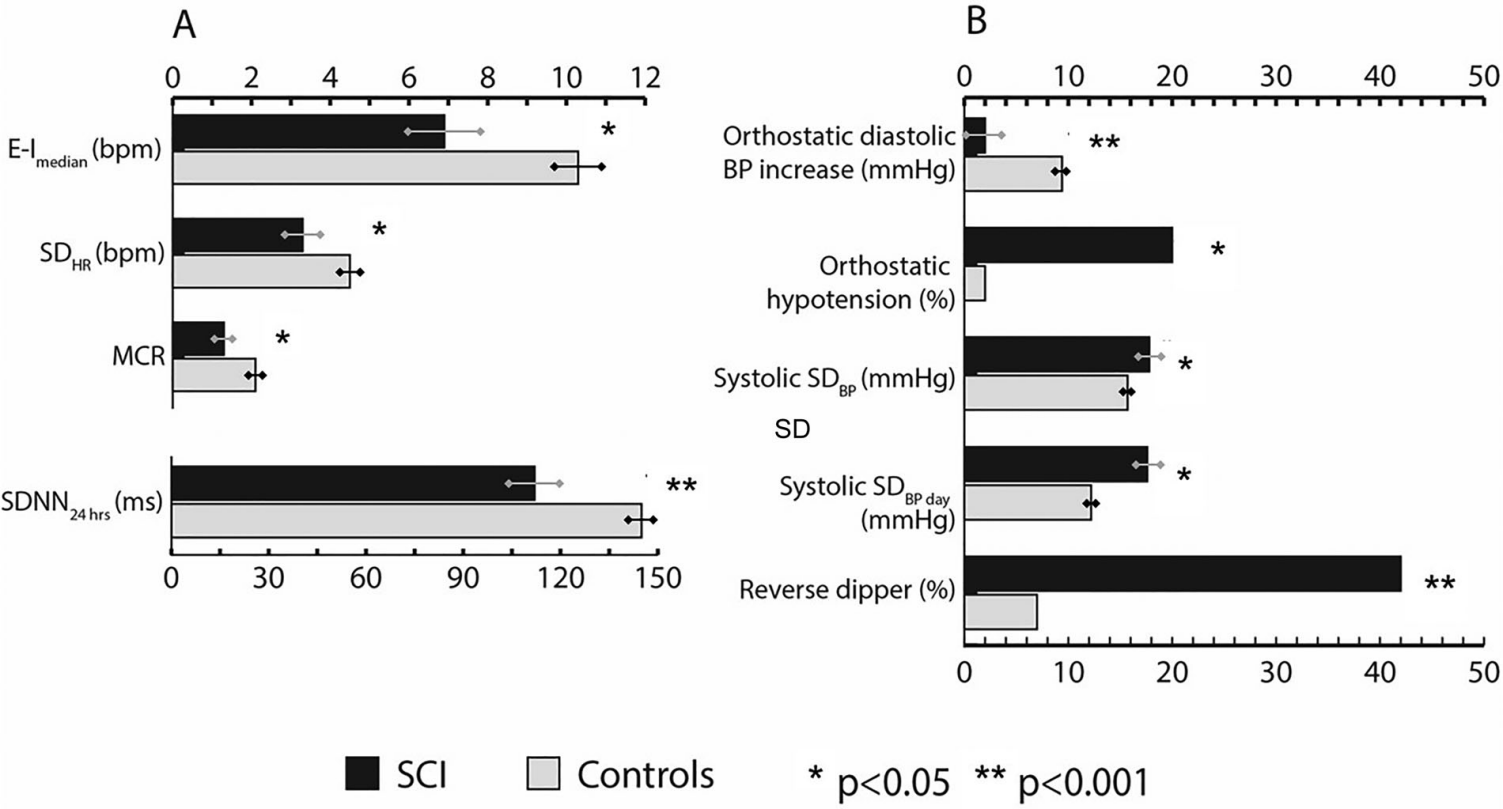
Key results (representing mean values and standard error of the mean; and proportions for orthostatic hypotension and reverse dipper) of the heart rate variability (A) and blood pressure (B) assessments. HRV results from deep breathing test except for SDNN (24h Holter). BP: blood pressure; E-I: expiration–inhalation difference; MCR: mean circular resultant; SCI: spinal cord injury; SD_BP_: standard deviation of blood pressure; SD_HR_: standard deviation of heart rate; SDNN: standard deviation of the NN interval.

In Table 3, descriptive findings of cardiovascular autonomic function and the conditional logistic regression analyses of the individuals with SCI and controls are presented. In Fig. 1, the key results of the HRV and BP assessments are illustrated.

**Table 3:** Descriptive findings of cardiovascular autonomic function and conditional logistic regression analyses of the 25 individuals with SCI and the 125 matched controls from the general population.

	Individuals with SCI	Matched controls	OR^a^ (95% CI)	P value
Electrocardiogram (mean ±SD; median, min-max)				
Deep breathing test^b^				
Heart rate (beats/min)	65 ±11; 64, 45–89	64 ±10; 64, 43–86	1.0 (0.96–1.05)	0.86
E-I_median_ (beats/min)	6.9 ±4.7; 4.9, 1.6–20.6	10.3 ±6.3; 8.9, 1.1–32.5	0.87 (0.77–0.97)	**0.017**
E/I (beats/min)	1.12 ±0.09; 1.09, 1.02–1.47	1.18 ±0.12; 1.14, 1.01–1.57	0.0 (0.0–0.34)	**0.023**
E-I_mean_ (beats/min)	7.9 ±5.1; 6.8, 2.0–20.9	11.2 ±6.1; 10.1, 1.5–29.7	0.88 (0.79–0.98)	**0.017**
SD_HR_ (beats/min)	3.3 ±1.9; 3.0, 0.8–8.2	4.5 ±2.5; 4.0, 0.5–14.4	0.75 (0.58–0.97)	**0.028**
MCR	1.3 ±0.9; 1.0, 0.05–3.3	2.1 ±1.3; 1.8, 0.21–6.8	0.43 (0.24–0.79)	**0.006**
RMSSD (ms)	39 ±40; 30, 7–204	53 ±39; 41, 5–200	0.99 (0.98–1.0)	0.14
24h Holter^c^				
Mean heart rate (beats/min)	73 ±12; 73, 49–101	76 ±10; 77, 51–102	0.96 (0.92–1.01)	0.12
Mean minimum heart rate (beats/min)	51 ±9; 49, 37–65	51 ±7; 50, 33–74	1.00 (0.94–1.06)	1.0
Mean maximum heart rate (beats/min)	116 ±21; 113, 76–157	140 ±18; 142, 101–183	0.92 (0.89–0.96)	**<0.001**
SDNN (ms)	112 ±34; 113, 55–172	145 ±38; 143, 56–262	0.97 (0.96–0.99)	**<0.001**
RMSSD (ms)	23 ±11; 22, 8–53	27 ±12; 24, 9–74	0.97 (0.93–1.02)	0.23
HF_day_ (ms^2^)	860 ±1000; 594, 125–5034	1073 ±896; 804, 62–4962	1.0 (1.0–1.0)	0.31
HF_night_^d^ (ms^2^)	1163 ±1226; 687, 19–4986	1630 ±1587; 1152, 34–9780		0.06
LF_day_^d^ (ms^2^)	2112 ±3421; 1423, 118–17562	2268 ±1464; 2161, 101–7661		0.10
LF_night_ (ms^2^)	2946 ±3103; 2030, 65–11280	3178 ±2252; 2881, 91–11725	1.0 (1.0–1.0)	0.66
LF/HF_day_	2.4 ±1.1; 2.4, 0.61–4.9	2.5 ±0.94; 2.4, 0.72–5.5	0.84 (0.49–1.42)	0.51
LF/HF_night_	2.9 ±1.7; 2.2, 0.38–6.2	2.5 ±1.3; 2.3, 0.63–7.5	1.25 (0.88–1.76)	0.21
LF/HF_day_ -LF/HF_night_	−0.48 ±1.0; −0.33, −3.2-1.1	0.008 ±0.87; 0.10, −3.2-2.9	0.54 (0.32–0.92)	**0.023**
Blood pressure (mean ±SD; median, min-max; n (%))				
Resting systolic BP (mmHg)	122 ±16; 121, 97–166	126 ±16; 123, 98–185	0.98 (0.95–1.01)	0.21
Resting diastolic BP (mmHg)	75 ±9; 73, 55–95	77 ±9; 77, 58–104	0.98 (0.93–1.02)	0.33
Orthostatic blood pressure^e^				
Difference in systolic BP (mmHg)	0.08 ±16; 3, −29-26	2.5 ±11; 2.5, −48-28	0.98 (0.94–1.02)	0.37
Difference in diastolic BP (mmHg)	2.0 ±10; 2.0, −29-21	9.4 ±7; 10, −18-24	0.89 (0.83–0.95)	**<0.001**
Orthostatic hypotension^f^	5 (20)	2 (2)	11 (2–59)	**0.004**
Ambulatory blood pressure^g^				
Mean systolic BP (mmHg)	111 ±14; 111, 90–145	125 ±11; 125, 101–159	0.89 (0.84-0.94)	**<0.001**
Mean systolic BP_day_ (mmHg)	112 ±15; 109, 95–148	129 ±12; 128, 106–158	0.89 (0.84-0.94)	**<0.001**
Mean systolic BP_night_ (mmHg)	108 ±16; 108, 78–140	117 ±12; 117, 86–161	0.95 (0.91-0.98)	**0.005**
Mean diastolic BP (mmHg)	67 ±8; 66, 50–90	77 ±8; 76, 56–100	0.84 (0.77-0.91)	**<0.001**
Mean diastolic BP_day_ (mmHg)	68 ±9; 67, 51–94	80 ±9; 79, 57–102	0.83 (0.77-0.91)	**<0.001**
Mean diastolic BP_night_ (mmHg)	63 ±9; 64, 48–83	70 ±9; 69, 51–98	0.92 (0.87-0.97)	**0.004**
Systolic SD_BP_ (mmHg)	17.8 ±4.7; 18.1, 11.9-28.9	15.7 ±4.5; 15.5, 6.5-30.5	1.13 (1.01-1.26)	**0.029**
Systolic SD_BP day_ (mmHg)	17.6 ±5.0; 16.9, 9.2-28.8	12.2 ±5.0; 13.7, 5.7-30.9	1.14 (1.04-1.25)	**0.006**
Systolic SD_BP night_ (mmHg)	13.4 ±6.0; 12.0, 5.2-26.9	11.6 ±4.9; 11.2, 3.1-30.5	1.06 (0.98-1.15)	0.13
Diastolic SD_BP_ (mmHg)	12.4 ±3.0; 12.5, 4.9-19.1	12.6 ±3.5; 12.3, 5.8-23.0	0.98 (0.86-1.12)	0.86
Diastolic SD_BP day_ (mmHg)	12.0 ±3.2; 11.8, 4.7-18.2	11.1 ±4.3; 10.2, 2.9-24.5	1.05 (0.95-1.17)	0.33
Diastolic SD_BP night_ (mmHg)	9.5 ±3.8; 8.2, 4.1-19.6	9.1 ±3.5; 8.6, 1.9-19.6	1.02 (0.91-1.16)	0.64
Dipper	5 (21)	47 (42)	0.37 (0.13-1.1)	0.07
Extreme-dipper	1 (4)	5 (5)	0.88 (0.10-8.1)	0.91
Non-dipper	8 (33)	52 (46)	0.56 (0.22-1.42)	0.22
Reverse-dipper	10 (42)	8 (7)	8 (3-23)	**<0.001**

Abbreviations: BP, blood pressure; E-I, expiration–inhalation difference; HF, high frequency; HRV, heart rate variability; LF, low frequency; MCR, mean circular resultant; NN, normal-to-normal, RMSSD; root mean square of successive differences, SBP; systolic blood pressure, SCI; spinal cord injury, SD_BP_, standard deviation of blood pressure, SD_HR_, standard deviation of heart rate, SDNN, standard deviation of the NN interval.

^a^ The ORs of the continuous variables implicate the odds of being an individual with SCI for a one-unit increase in the variable

^b^ Number of individuals with SCI and controls 25 and 103, respectively.

^c^ Number of individuals with SCI and controls 25 and 122, respectively. HRV data of one individual with SCI (atrial fibrillation) and eight controls (three due to atrial fibrillation and five corresponding to the excluded individual with SCI) were excluded.

^d^ Conditional logistic regression computed on transformed data due to skewness.

^e^ Number of individuals with SCI and controls 25 and 112, respectively

^f^ Defined as a drop in systolic BP≥20 or diastolic BP ≥10 mmHg.

^g^ Number of individuals with SCI and controls 24 and 117, respectively. Night measures were missing in five additional controls.

DBT variables were significantly lower among the individuals with SCI than the controls, except for RMSSD and HR. The 24h Holter assessments demonstrated, among the individuals with SCI compared with the controls, significantly lower mean maximum HR, lower SDNN and lower LF/HF ratios during day compared with night. The mean difference between day and night LF/HF ratios among the individuals with SCI was significant (−0.48; 95% CI −0.9, −0.05; P = 0.015).

Resting BP was comparable, whereas diastolic orthostatic BP increase was significantly lower among the individuals with SCI than among the controls (2.0 vs 9.4 mmHg; P < 0.001). Five individuals with SCI (20%) and two controls (2%) were identified as having orthostatic hypotension (OR = 11; 95% CI 2-59). ABPM showed, among the individuals with SCI, significantly lower mean systolic and diastolic BP measures, whereas the systolic standard deviation of BP (SD_BP_) was significantly higher except during the night. A rise in mean systolic BP during the night occurred in 42% and 7% of the individuals with SCI and controls, respectively (OR = 8; 95% CI 3-23).

### Associations between NLI and cardiovascular autonomic function

In Table 4, significant associations between NLI and cardiovascular autonomic function are presented. The remaining non-significant associations are presented as supplementary material. Higher NLI was negatively associated with four DBT variables, maximum HR, LF/HF day and night ratios, and resting BP. Higher NLI was positively associated with systolic SD_BP_.

**Table 4:** Spearman rank correlation between neurological level of injury and cardiovascular autonomic function of the 25 individuals with SCI.

Variable	Correlation coefficient	P value
Deep breathing test		
Heart rate (beats/min)	−0.40	0.048
E-I_mean_ (beats/min)	−0.42	0.037
SD_HR_ (beats/min)	−0.48	0.015
MCR	−0.41	0.041
24h Holter		
Maximum heart rate (beats/min)	−0.63	<0.001
LF/HF_day_	−0.53	0.008
LF/HF_night_	−0.45	0.028
Blood pressure		
Resting systolic BP (mmHg)	−0.40	0.048
Ambulatory blood pressure		
Systolic SD_BP day_ (mmHg)	0.46	0.030

Presenting only statistically significant correlations and defined as each higher neurological level of injury C1-T6.

Abbreviations: BP, blood pressure; E-I, expiration–inhalation difference; HF, high frequency; LF, low frequency; MCR, mean circular resultant, SCI, spinal cord injury; SD_BP_, standard deviation of blood pressure; SD_HR_, standard deviation of heart rate.

## Discussion

In the present study, cardiovascular autonomic function was comprehensively assessed in middle-aged people with long-term cervical and upper thoracic SCI, and matched controls from the general population. The comprehensive cardiovascular assessment of long-term SCI is considered a novelty. HRV among the individuals with SCI was significantly lower than among the controls. Additionally, individuals with SCI had significantly lower diastolic orthostatic BP and significantly higher BPV than the controls. Ambulatory 24h assessments demonstrated significantly different cardiovascular circadian patterns among the individuals with SCI than among the controls. A higher NLI was significantly and negatively correlated with several HRV variables, and significantly and positively correlated with BPV. Thus, we can confirm our hypotheses that cardiovascular autonomic function is significantly impaired among the individuals with SCI compared with the controls, and that a higher NLI is related to cardiovascular autonomic impairments.

### Heart rate variability

DBT was used to assess the autonomic response causing respiratory sinus arrythmia, which is primarily due to parasympathetic activity (2). The reduced expiration-inhalation differences thus indicate impaired cardiovagal function. In people with SCI, the vagal innervation is preserved, and the results may be due to differences in sympatho-vagal balance and/or altered respiratory mechanics affecting the HR (6).

The 24h ECG registrations demonstrated reduced HRV as measured by SDNN, reflecting both parasympathetic and sympathetic activity, whereas RMSSD, reflecting parasympathetic activity, was comparable between individuals with SCI and controls. This finding can be expected due to reduced supraspinal sympathetic activity among the individuals with SCI. The comparable LF/HF ratios may indicate that there is still a sympatho-vagal balance among the individuals with SCI (6). However, the significant negative relationship between NLI and LF/HF ratios indicates that this is partly depending on NLI, with decreasing sympathetic dominance with higher NLI.

DBT and 24h HRV results must be interpreted from generated knowledge from the general non-SCI population, which may not be generalizable to the SCI population. However, previous research indicates pathophysiological links between reduced HRV and metabolic abnormalities, dysrhythmias, and an influence on atherosclerotic plaque progression (2). SDNN values <100 on a 24h ECG has been associated with increased mortality in the general population (6). The mean SDNN (112) among the individuals with SCI is still above this limit, although lower than in the controls (145).

Differences in physical activity may have an impact on the HRV results. Among the individuals with SCI there were four individuals who engaged in regular leisure time physical activity and 13 who reported a sedentary leisure time (4). Physical activity has been reported to improve HRV and therefore it is likely that physical inactivity among the individuals with SCI made the differences greater (19).

Several other factors exert short-term and long-term effects on HRV. Significant contributors to HRV such as ventilation, baroreflexes and renin-angiotensin-aldosterone-system are commonly affected by SCI (6,20–22). Consequently, there is a need to determine what factors contribute to reduced HRV in SCI, and the prognostic significance of these impairments.

A few studies have reported results from 24h HRV in people with SCI (23–27). The RMSSD results in the present study are in agreement with those of Rosado-Rivera *et al.* (23) who assessed 30 individuals (mean age 43 years) with cervical and upper thoracic SCI and 10 controls (23). In the study by Wang *et al.* (24), 12 individuals with low paraplegia and 16 individuals with tetraplegia (mean ages 35 and 36 years, respectively) were assessed with 24h HRV. The authors reported similarly significantly lower SDNN as in the present study but higher non-significant LF/HF ratios among the participants with tetraplegia than among those with paraplegia. The LF/HF ratio was decreasing with higher NLI in the present study. In the study by Wang *et al.* (24), the participants with paraplegia had NLI T10-L1 which may explain this difference. These individuals probably have a cardiac sympathetic functional integrity and therefore the sympatho-vagal balance may be different. Another study by Bunten *et al.* (25) reported 24h HRV results in six individuals with tetraplegia and seven individuals with paraplegia (mean age 44 and 49 years, respectively and unknown injury duration) and compared with 13 controls. No significant differences were found in the time domain analyses. Frequency domain analyses revealed significantly lower LF among the participants with tetraplegia than among controls. Ruangsuphaphichat *et al.* (26) investigated test-retest reliability of 24h HRV in 45 individuals with SCI (18 with NLI above T6, mean age 48 years and median five years after SCI). Among the 18 individuals, they reported lower median HF (467) and LF (549) power, whereas median SDNN and RMSSD were similar, as compared to the present study. Our results are in agreement with Saengsuwan *et al.* (27) who explored diurnal variations of HRV in 58 individuals (22 with NLI above T6, median age 51, and median five years after SCI). That study reported increases in HF and LF power during the night as compared to daytime (27). These, to some extent inconsistent, results may be due to that the target groups and analyses were different as compared to the present study, and the small sample sizes. For example, Bunten *et al.* (25) compared individuals with tetraplegia exclusively, whereas the present study compared both individuals with tetraplegia and high paraplegia. Furthermore, Ruangsuphaphichat *et al.* (26) reported results from a study population that to a larger extent comprised individuals with paraplegia and incomplete SCI.

Taken together, our results indicate that cardiac autonomic risk markers for CVD (2,6) are prevalent in this population. Larger studies are needed to further explore relations between SCI and HRV in people with long-term SCI.

### Blood pressure variability and orthostatic blood pressure

Resting BP was comparable between individuals with SCI and controls, which was not the case for the 24h measurements. These were significantly lower among the individuals with SCI, possibly because resting BP was measured in the supine position. In the supine position, there will be a redistribution of the effective blood volume generating a greater cardiac output and a higher BP among the individuals with SCI (28). In the seated position during most of the 24h assessments, gravity in combination with impaired vascular capacitance regulation following loss of supraspinal sympathetic control results in an accumulation of blood in the lower body leading to a lower BP (28). In addition, previously described differences in physical activity may also have an impact on these differences as both the physiological BP increase during physical activity is diminished, and the pressor response is impaired among the individuals with SCI (4,29). The previously described hemodynamic effect, in combination with impaired sympathetic regulation of the peripheral resistance, probably causes the reduced diastolic orthostatic BP increase among the individuals with SCI as compared with the controls (3,28).

People with normal resting BP and increased 24h BP have more organ damage, and accordingly the lower 24h assessments among middle-aged people with cervical and upper thoracic SCI (30) may be protective. This is corroborated by our previous study of atherosclerosis demonstrating no increase in atherosclerotic burden among the individuals with SCI compared with controls (13).

The presence of orthostatic hypotension was lower in the present study (20%) than in a younger population (mean 40 years) of 19 individuals with cervical and upper thoracic SCI (47%) (31). It may be due to the longer time since injury in our study as orthostatic hypotension is regarded to stabilize over time (21). This may be due to adaptations following injury such as renin-angiotensin-aldosterone-system activity (32).

The individuals with SCI had significantly higher systolic BPV (SD_BP_) than the controls. The increased BPV is possibly caused by the known labile BP due to loss of supraspinal sympathetic control of the BP (33). Thus, people with cervical and upper thoracic SCI experience both hypertension due to AD and hypotension causing the BPV (33). Our results are in agreement with the study of Wang *et al.* (33) assessing 24h BP instability in 33 participants (mean age 35 years) with cervical and upper thoracic SCI with diagnosed orthostatic hypotension and comparing with ambulatory and non-ambulatory controls. In the present study, the individuals with SCI and controls did not keep a diary which is suggested in future research to support the interpretation of the results and to identify the magnitude of autonomic responses.

Taken together, our results indicate that among people with cervical and upper thoracic SCI also vascular risk markers for CVD are prevalent (34,35), but the lower 24h BP may be a protective factor for CVD.

### Cardiovascular circadian patterns

The 24h ECG registrations revealed circadian patterns of both BP and HRV. We found that during the night, the LF/HF ratio increased, and a rise in systolic BP frequently occurred among the individuals with SCI compared with the controls. The increase in LF/HF ratio during the night was also observed among the participants with tetraplegia in the study by Wang *et al.* (24). The differences in circadian pattern of HRV are noticeable but the pathophysiology needs to be determined. These circadian patterns may be the result of the altered hemodynamics of SCI previously described. In the supine position during the night, gravitational forces are negated, and blood will be redistributed to the central circulation from capacitance veins (28). The increase in effective blood volume increases the cardiac filling and, according to the Frank-Starling law, results in a higher BP (28). Accordingly, an increase in BP will increase the activity of baroreceptors (6). In the resting condition, baroreflex activity reflects the LF band which therefore may explain the increased LF/HF ratio (6). Among the individuals with SCI, the respiratory mechanics in the seated vs supine position are different and this may also exert an effect on day and night HRV differences (20). Another potentially contributing factor is the highly prevalent sleep disordered breathing among the individuals with SCI (4) which may impact the LF/HF ratio and BP (36). Nocturnal differences in HRV have been reported to reflect different sleep stages (37). This has been speculated to impact the circadian pattern of CVD events occurring more frequently in the morning due to differences in sympatho-vagal balance (37). The nocturnal differences in HRV may therefore have a prognostic significance in people with SCI.

We found that 42% of the individuals with SCI exhibited a nocturnal systolic BP rise, a reverse-dipper pattern of BP, previously shown in cervical and upper thoracic SCI (33,38). The reverse-dipper pattern is additionally a CVD risk marker that seems to be highly prevalent in this population (38,39).

### NLI and cardiovascular impairment

The relationships between NLI and several of the HRV variables, and BPV are new and noteworthy. Associations with HRV and BPV can be expected due to the segmental organization of the cardiopulmonary innervation above T6. Both cardiovascular (sympathetic activity) and pulmonary innervation (ventilatory capacity) are reduced in relation to a more rostral NLI which may influence the results. There is one important difference though. The sympathetic outflow is thoracolumbar and, therefore, in individuals with tetraplegia, all sympathetic outflow may be affected, whereas ventilatory capacity is segmental also in cervical SCI (3,20). Taken together, this emphasizes the need for future research efforts to elucidate the mechanisms of the presented cardiovascular impairments.

### Clinical implications

The interpretation of the clinical implications of the present study warrants caution due to the small sample. We identify that factors which may increase the risk for CVD are prevalent. Therefore, preventive strategies, such as physical activity are emphasized at follow-ups. The clinical assessment and management of cardiovascular functional impairments are of importance. The 24h ECG and ABPM assessments are feasible and considered valuable clinical tools to determine treatment strategies. These can be used in combination with a diary to understand mechanisms and consequences of BPV in people with SCI, for example BP responses during transfers and bowel routines.

### Strengths and limitations

A major strength of this study was the comprehensiveness of the included assessments enabling a broad and in-depth assessment of the cardiovascular autonomic function. The individuals with SCI and controls have been demonstrated to comprise a representative sample (13). The same staff performed all assessments, over the same period of time and with the same equipment for both the individuals with SCI and the matched controls. Moreover, a detailed comparison between individuals with SCI and the general population could be done due to the careful matching procedure. A larger sample size, however, would have allowed more detailed inferences and a longitudinal design would have allowed inferences about aging *per se*. Respiratory parameters such as tidal volume were not controlled for. This may have increased differences between individuals with SCI and controls and the relationship between NLI and cardiac parameters (6,40). The different testing procedures of orthostatic responses is another limitation, but it was not possible for the individuals with SCI to follow the protocol of SCAPIS. The hemodynamic stress was greater among the controls due to a stand-up test compared with the individuals with SCI performing at sit-up test. Therefore, the reported differences may be underestimated. Among the individuals with SCI, the frequent use of medications with anticholinergic effects (4), possibly caused greater differences between individuals with SCI and controls as anticholinergic activity reduces HRV (41). However, we should not expect a major impact as the NLI was related to HRV variables and the exposure of anticholinergic medications was not related to the NLI. Finally, comorbidities may also influence the outcome. Diabetes, which occurred more frequently among the individuals with SCI, has a negative influence on HRV variables (42), whereas smoking, which was more frequent among the controls, may have the opposite effect (43). Differences in BMI are more uncertain as BMI underestimates obesity in SCI due to body compositional changes (44). We do not expect a major influence as the WC was similar. Taken together, the potential effects of these uncontrolled factors warrant caution when interpreting the results.

## Conclusions

This exploratory study has comprehensively assessed cardiovascular autonomic function in middle-aged people with cervical and upper thoracic SCI, with a mean time since injury of 28 years. The results were compared with matched controls from the general population. HRV, orthostatic BP responses and BPV were significantly impaired, and circadian patterns of HRV and BP were significantly different compared with the general population. Impairments of HRV and BPV were more pronounced in relation to a higher NLI. These cardiovascular autonomic impairments may be independent risk markers for CVD in this population. Future research is needed to understand the pathophysiological mechanisms underlying these results. In the longer term, the prognostic significance of cardiovascular autonomic impairments in the aging population with SCI needs to be evaluated in order to improve risk assessment of CVD in SCI.

## Supplementary Material

Table 5.docx

## Data Availability

All data were archived according to the Swedish Act concerning the Ethical Review of Research Involving Humans to attain confidentiality and are available on reasonable request.

## Disclaimer statements

**Contributors** None.

**Funding** SPICA is supported by a PhD position grant from the Promobilia Foundation [grant number F18515], and by project grants from the Promobilia Foundation, the Norrbacka-Eugenia Foundation, Skåne County Council Research and Development Foundation, Skåne University Hospital (SUS Stiftelser och Donationer), Stiftelsen för bistånd åt rörelsehindrade i Skåne, and NEURO Sweden. The main funding body of The Swedish CArdioPulmonary bioImage Study (SCAPIS) is the Swedish Heart-Lung Foundation. The SCAPIS is also funded by the Knut and Alice Wallenberg Foundation, the Swedish Research Council and VINNOVA (Sweden’s Innovation agency), the University of Gothenburg and Sahlgrenska University Hospital, the Karolinska Institute and Stockholm County Council, Linköping University and Linköping University Hospital, Lund University and Skåne University Hospital, Umeå University and Umeå University Hospital, Uppsala University and Uppsala University Hospital. DBT ECG processing and analysis was supported by The Swedish Heart-Lung Foundation [grant number 20200674].

**Declaration of interest** None.
