# Supplementary material for: Cardiovascular autonomic function in middle-aged people with long-term cervical and upper thoracic spinal cord injuries
Source: J Spinal Cord Med. 2024 Oct 11;48(3):447–60. doi: 10.1080/10790268.2024.2403791 (PMC12035942; doi:10.1080/10790268.2024.2403791)
Supplement: Table 5.docx [file YSCM_A_2403791_SM4839.docx]

| Table 5. Spearman rank correlation between neurological level of injury and cardiovascular autonomic function of the 25 individuals with SCI | | |
| --- | --- | --- |
| Variable | Correlation coefficient | P-value |
| Deep breathing test |  |  |
| E/I (beats/min) | -0.23 | 0.26 |
| E-I_median_ (beats/min) | -0.34 | 0.09 |
| RMSSD (ms) | 0.04 | 0.85 |
| 24hrs Holter |  |  |
| Mean heart rate (beats/min) | -0.40 | 0.05 |
| Minimum heart rate (beats/min) | -0.27 | 0.20 |
| SDNN (ms) | -0.24 | 0.25 |
| RMSSD (ms) | 0.22 | 0.30 |
| Blood pressure |  |  |
| Resting diastolic BP (mmHg) | -0.09 | 0.66 |
| Orthostatic blood pressure^e^ |  |  |
| Difference in systolic BP (mmHg) | 0.32 | 0.12 |
| Difference in diastolic BP (mmHg) | 0.10 | 0.62 |
| Ambulatory blood pressure |  |  |
| Mean systolic BP (mmHg) | -0.17 | 0.43 |
| Mean systolic BP_day_ (mmHg) | -0.24 | 0.26 |
| Mean systolic BP_night_ (mmHg) | -0.04 | 0.85 |
| Mean diastolic BP (mmHg) | -0.08 | 0.70 |
| Mean diastolic BP_day_ (mmHg) | -0.11 | 0.62 |
| Mean diastolic BP_night_ (mmHg) | 0.02 | 0.94 |
| Systolic SD_BP_ (mmHg) | 0.32 | 0.13 |
| Systolic SD_BP night_ (mmHg) | 0.27 | 0.21 |
| Diastolic SD_BP_ (mmHg) | 0.14 | 0.53 |
| Diastolic SD_BP day_ (mmHg) | 0.18 | 0.41 |
| Diastolic SD_BP night_ (mmHg) | 0.25 | 0.23 |
| Presenting only statistically non-significant correlations and defined as each higher neurological level of injury C1-T6  Abbreviations: BP=blood pressure; E-I=expiration–inhalation difference; NN=normal-to-normal; RMSSD=root mean square of successive differences; SBP=systolic blood pressure; SCI=spinal cord injury; SD_BP_=standard deviation of blood pressure | | |
